# Preparation and Imaging Investigation of Dual-targeted C_3_F_8_-filled PLGA Nanobubbles as a Novel Ultrasound Contrast Agent for Breast Cancer

**DOI:** 10.1038/s41598-018-21502-x

**Published:** 2018-03-01

**Authors:** Jing Du, Xiao-Yu Li, He Hu, Li Xu, Shi-Ping Yang, Feng-Hua Li

**Affiliations:** 1 Department of Ultrasound, Renji Hospital, School of Medicine, Shanghai Jiao Tong University, 160 Pu Jian Rd, Shanghai, 200127 China; 2 Department of Biomedical Engineering, Case Western Reserve University Schools of Medicine, 10900 Euclid Ave., Cleveland, OH 44106 USA; 3 Department of Chemistry, Shanghai Normal University, 100 Gui Lin Rd, Shanghai, 200234 China

## Abstract

Molecularly-targeted contrast enhanced ultrasound (US) imaging is a promising imaging strategy with large potential for improving diagnostic accuracy of conventional US imaging in breast cancer detection. Therefore, we constructed a novel dual-targeted nanosized US contrast agent (UCA) directed at both vascular endothelial growth factor receptor 2 (VEGFR2) and human epidermal growth factor receptor 2 (HER2) based on perfluoropropane (C_3_F_8_)-filled poly(lactic-co-glycolic acid) (PLGA) (NBs) for breast cancer detection. *In vitro*, single- or dual-targeted PLGA NBs showed high target specificities and better effects of target enhancement in VEGFR2 or HER2-positive cells. *In vivo*, US imaging signal in the murine breast cancer model was significantly higher (*P* < 0.01) for dual-targeted NBs than single-targeted and non-targeted NBs. Small animal fluorescence imaging further confirmed the special affinity of the dual-targeted nanosized contrast agent to both VEGFR2 and HER2. Immunofluorescence and immunohistochemistry staining confirmed the expressions of VEGFR2 and HER2 on tumor neovasculature and tumor cells of breast cancer. In conclusions, the feasibility of using dual-targeted PLGA NBs to enhance ultrasonic images is demonstrated *in vitro* and *in vivo*. This may be a promising approach to target biomarkers of breast cancer for two site-specific US molecular imaging.

## Introduction

Molecular imaging can visualize the biological processes at the molecular and cellular levels *in vivo* using certain tracers for specific molecular targets. Molecular imaging of breast cancer can be performed with various imaging modalities, such as magnetic resonance imaging (MRI) using a contrast agent, positron emission tomography (PET) using a positron-emitting radionuclide, single photon emission computed tomography (SPECT) using a gamma-emitting radionuclide, optical imaging using a fluorescent dye, or contrast-enhanced ultrasound (US) using a contrast agent^1^. However, US molecular imaging could combine the advantages of conventional US with the capabilities of molecular imaging to visualize molecular signatures with high sensitivity and specificity *in vivo*^2^. Molecularly-targeted contrast enhanced US imaging is a promising imaging strategy with large potential for improving diagnostic accuracy of conventional US imaging in breast cancer detection^2–4^. Along with the rapid development of contrast-enhanced US technology and bio-nanotechnology, a mass of targeted US contrast agents (UCAs) have emerged^5^. The non-invasive US molecular imaging without ionizing radiation enables sensitive and specific depiction of molecular targets of breast cancer with the use of targeted UCAs.

Targeted UCAs represented by gas-filled microbubbles (MBs) are stabilized by a shell. The shell can be further functionalized by adding various types of ligands to make MBs attach to receptors that are differentially expressed on the neovasculature of different cancer types including breast cancer^6^. Poly(lactic-co-glycolic acid) (PLGA) MBs are the most common type of UCAs available at present. The polymeric shell improves the stability of the capsules, compared to that of those stabilized by a monomolecular layer of surfactant^7,8^. Furthermore, PLGA microcapsules contain gas, which increases their scattering power and leads to a high echogenicity due to the high compressibility and low density of the gases^8^. PLGA hollow or porous MBs have already been proved to be an efficient UCA in previous researches^9,10^. They can also be modified with monoclonal antibody or polypeptide for targeting US or dual-mode US/MR molecular imaging^11,12^. However, PLGA MBs with the size of several microns can not effectively penetrate the leaky tumor vasculature to target the cancer cells, since the fenestrate openings of typical tumors are within the range of 400 nm–600 nm^13^. Therefore, the development of nanobubble-based UCAs, which can permeate through the tumor vasculature, is urgently required.

In recent years, nanobubbles (NBs) with various shells composed of polymers or phospholipids and cores (gas, liquid or solid) have been applied in extravascular ultrasonic imaging and exhibit good contrast enhancement^14^. Polymer-shelled NBs can access the extravascular space and provide unique advantages for targeted specific US imaging due to their small size, novel physical and surface properties. However, only a few studies have developed nanobubble-based specific US imaging agents for improved detection and diagnosis for breast cancer^15,16^.

Vascular endothelial growth factor receptor 2 (VEGFR2) is a best-characterized molecular marker of tumor angiogenesis, and is overexpressed on tumor endothelial cells during tumor angiogenesis. VEGFR2 is an endothelium specific receptor tyrosine kinase, and activation of the VEGF/VEGFR2 axis triggers multiple signaling networks that result in endothelial cell survival, mitogenesis, migration, and differentiation, as well as altered vascular permeability^17^. Several studies have shown that US molecular imaging using VEGFR2-targeted MBs allows highly accurate detection of breast cancer, even ductal carcinoma *in situ* (DCIS)^18–20^.

Human epidermal growth factor receptor 2 (HER2), also known as receptor tyrosine kinase erbB-2, is a member of the transmembrane epidermal growth factor receptor family^21,22^. It causes the activation of different downstream cascades, including the mitogen-activated protein kinase (MAPK) proliferation pathway and the phosphoinositide 3-kinase (PI3K/Akt) prosurvival pathway^23^. HER2 positive expression is found in 25–30% of breast cancers and is associated with aggressive tumor behavior, higher rate of recurrence and decreased survival^1,24^. HER2-targeted molecular imaging is attracting great interest and may become an important method for early breast cancer detection. Recent researches have described HER2-targeted US, MRI and PET-CT contrast agents for improved detection and diagnosis of breast cancer^1,15,16,24^.

To achieve both high sensitivity and specificity in detecting breast cancer with US molecular imaging, it is of paramount importance to identify dual or multiple biomarkers as potential molecular imaging targets that are differentially expressed on the neovasculature and cancer cells compared to normal tissue and benign breast lesions. Due to their higher avidity that comes from the dual or multi-ligand approach, the dual or multi-targeted US MBs generate significantly higher acoustic signals compared to the single-targeted counterparts^17,25^. The benefits of multi-targeting strategies are consistent with some previous reports of multi-ligand functionalized particle design for cell targeting and drug delivery^26,27^. In the previous studies, the dual-targeted UCAs were mostly micro-sized, and the dual-targeted nanosized UCAs that target simultaneously the specific receptors in tumor neovasculature and breast cancer cells, had not yet been reported. Furthermore, to the best of our knowledge, no study has systematically investigated the potentials of dual-targeted PLGA NBs for *in vivo* US molecular imaging of breast cancer.

The goal of this study was to develop a novel dual-targeted UCA directed at both VEGFR2 and HER2 based on PLGA NBs for breast cancer detection. Accordingly, the present study was designed: (i) to prepare perfluoropropane (C_3_F_8_)-filled NBs with a biodegradable polymeric shell composed of PLGA and (ii) to test the feasibility of using C_3_F_8_-filled PLGA NBs modified with two different types of targeting molecules to specifically target surface receptors of tumor neovasculature and cancer cells, providing ultrasonic enhancement upon targeting. Such dual-ligand targeting strategy is based on the fact that VEGFR2 is overexpressed only in the newly formed tumor blood vessels rather than in the static ones of normal tissues, which makes it an ideal target for detection and antiangiogenesis therapy of solid tumors. Meanwhile, HER2 modification may further enhance the accumulation of the NBs in tumor tissue via specific interaction with breast cancer cells.

## Materials and Methods

### Materials

PLGA (50:50; molecular weight, 40,000) was obtained from Jinan Daigang Biomaterial Co., Ltd. (Shandong, China). Polyvinyl alcohol (PVA; 88% mole hydrolyzed) and (D+)-camphor were purchased from Aladdin Chemistry Co., Ltd (Shanghai, China). Methylene chloride, isopropanol, mannitol and phosphotungstic acid were purchased from Sinopharm Chemical Reagent Co., Ltd (Shanghai, China). 1-ethyl-3-(3-dimethylaminopropyl)-carbodiimide (EDC) and N-hydroxysuccinimide (NHS) were purchased from Sigma–Aldrich (St. Louis, MO, USA). Fluorescein isothiocyanate (FITC)-conjugated rabbit anti-human HER2 monoclonal antibody and control rat immunoglobulin G (IgG) antibody were synthesized by BD Biosciences (Palo Alto, CA, USA). Phycoerythrin (PE)-conjugated rat anti-mouse VEGFR2 monoclonal antibody was synthesized by Shanghai Jinmai Biotechnology Co., Ltd (Shanghai, China). C_3_F_8_ gas was purchased from Shanghai Renjie Ling Optics Instrument Co., Ltd (Shanghai, China).

### Cell culture

Human breast cancer cell lines SKBR3 and MDA-MB-231 were obtained from the Cell Bank of the Chinese Academy of Sciences (Shanghai, China). The cells were cultured in Dulbecco’s modified Eagle medium (DMEM) supplemented with 10% fetal bovine serum (FBS), 1% L-glutamine and 1% penicillin-streptomycin. Mouse angiosarcoma SVR cells was purchased from American Type Culture Collection (ATCC) (Rockville, MD, USA) and cultivated in Dulbecco’s modified Eagle’s medium with a high concentration of glucose (4.5 g/L) and L-glutamine (Invitrogen, USA), and supplemented with 10% FBS and penicillin (100 U/mL) and streptomycin (100 μg/mL). Mouse breast cancer 4T1 cells were purchased from the Cell Bank of the Chinese Academy of Sciences (Shanghai, China). 4T1 cells were cultured in RPMI-1640 cell culture media (HyClone Laboratories, Inc., Logan, UT, USA) and appended with 10% FBS. The cell cultures were maintained in a humidified atmosphere of 5% CO_2_ at 37 °C with the medium changed every other day.

### Animals

Nude female Balb/c mice (4–6 weeks old, body weight: 20 ± 1.5 g) were supplied by the Shanghai Slack Laboratory Animal Center (Shanghai Laboratory Animal Co., Ltd, Shanghai, China). All animals were housed in accordance with the Guide for the Care and Use of Laboratory Animals adopted by the National Institutes of Health, and all procedures were approved by the Institutional Animal Care and Use Committee, which was certified by the Shanghai Association for Accreditation of Laboratory Animal Care (Shanghai, China).

### Preparation of C_3_F_8_-filled PLGA NBs

PLGA NBs were prepared using an adapted oil-in-water emulsion solvent evaporation process according to previous reports^28,29^. PLGA (125 mg) and camphor (12.5 mg) were dissolved in methylene chloride (3.5 mL), and then the O/W emulsion was generated by adding the oil phase dropwise (7.0 mL/h) to the PVA aqueous solution (2% w/v, 20 mL). The system was then emulsified in an ice bath using a probe-type sonifier (JY92‐II ultrasonic processor; Ningbo Scientz Biotechnology Co., Ltd, China) at 650 W for 180 s, in a pulse mode with sonication turned off for 4 s and on for 2 s. After the emulsion, the suspension was added to 100 mL isopropanol solution (5% v/v) and stirred for 5 h by a magnetic stirrer to extract the residual methylene chloride. Then NBs were collected by centrifugation (16,000 rpm, 10 min) and washed three times with deionized water to remove free-floating PVA. Finally, the NBs were freeze-dried, and C_3_F_8_ gas was introduced into the lyophilization chamber through a specialty, vial connector, with the flow rate of 50 mL/min for 1 min. Then, the screw vials were capped.

### Preparation of targeted PLGA NBs

FITC-conjugated anti-HER2 monoclonal antibody and PE-conjugated anti-VEGFR2 monoclonal antibody were covalently linked to the C_3_F_8_-filled PLGA nanobubble surface using a carbodiimide technique. 13 mg PLGA NBs was resuspended in 6.5 mL phosphate buffer saline (PBS), and incubated with 1 mL of 400 mM EDC and 1 mL of 100 mM NHS for 30 min at room temperature with gentle stirring. The suspension was centrifuged at 16,000 rpm for 10 min at 25 °C and washed with deionized water three times to obtain purified PLGA NBs. Then, the precipitate was redispersed into 5 mL PBS. To prepare dual-targeted PLGA NBs (NB_D_), 2.4 mL of the activated PLGA NBs were incubated with FITC-conjugated anti-HER2 monoclonal antibody (240 μL, 0.1 mg/mL) and PE-conjugated anti-VEGFR2 monoclonal antibody (120 μL, 0.2 mg/mL) in thermostatic oscillator for 60 min. Similarly, single HER2 or VEGFR2-targeted PLGA NBs (NB_H_ and NB_V_) were obtained. 1.2 mL of the activated PLGA NBs were separately incubated with FITC-conjugated anti-HER2 monoclonal antibody (120 μL, 0.1 mg/mL) or PE-conjugated anti-VEGFR2 monoclonal antibody (60 μL, 0.2 mg/mL) in thermostatic oscillator for 60 min. Nontargeted NBs (NB_C_) conjugated with control rat IgG antibody were also prepared. Non-specific rat IgG isotype (120 μL, 0.1 mg/mL) was added to 1.2 mL of the activated PLGA NBs using the same procedure described above. The resulting antibody–nanobubble bioconjugates were centrifuged (16,000 rpm, 10 min) and washed three times with PBS to remove any remaining unbound antibodies. The precipitate was redispersed into the same volume of PBS as the initial volume of the activation solution.

### Characterization of NBs

The surface morphology of C_3_F_8_-filled PLGA NBs was investigated using an S-4800 field emission scanning electron microscope (FE-SEM; Hitachi, Tokyo, Japan). The samples of C_3_F_8_-filled PLGA NBs were dispersed in deionized water, spread over a piece of aluminum foil and dried at room temperature. The samples were subsequently sputter coated with a layer of gold using a fine coat ion sputter (JFC-1100; JEOL Ltd, Tokyo, Japan) prior to FE-SEM imaging.

A JEOL JEM-2100 high-resolution transmission electron microscope (HR-TEM; Hitachi, Tokyo, Japan) was used to observe the morphology and structure of C_3_F_8_-filled PLGA NBs. The PLGA nanobubble suspension was dropped onto a formvar film-coated copper grid for 2 min. The samples were further stained with 1% (w/v) phosphotungstic acid for 2 min and placed on copper grids for viewing by HR-TEM to confirm the absence of the hollow structure.

The samples were dispersed in deionized water to obtain a uniform suspension. The size distributions and Zeta potentials of the NB_C_, NB_H_, NB_V_ and NB_D_ were evaluated three times for each sample using dynamic light scattering (Zetasizer Nano ZS model ZEN3690; Malvern Instruments, Ltd., Malvern, UK).

### *In vitro* receptor-specific targeting studies

In this study, SKBR3, a human breast cancer cell line expressing abundant HER2 on the cell membrane was used as the positive cell line. MDA-MB-231, another breast cancer cell line with low HER2 expression, was used as the negative cell line for testing HER2-mediated specificity of nanobubble targeting. In addition, mouse angiosarcoma SVR cells with high VEGFR2 expression were used as the positive cell line for confirming the specific binding of targeted NBs to VEGFR2. Mouse breast cancer 4T1 cells with negative VEGFR2 expression were used as a negative control.

Fluorescent staining was performed to verify the targeting of the NBs onto cell surfaces. Individual samples of SKBR3, MDA-MB-231, SVR and 4T1 cells were seeded in 24-well plates at a density of 1 × 10^5^ cells per well. The medium was removed by aspiration, and the cells were washed twice with PBS. They were divided into five groups as follows: the simple cells as control group, the non-targeted control group, the single HER2-targeted group, the single VEGFR2-targeted group and the dual-targeted group. Four kinds of cells in the non-targeted control group were treated with 100 μL PLGA NB_C_ and the cells in the dual-targeted group were treated with 100 μL NB_D_. For the single HER2-targeted group, SKBR3 and MDA-MB-231 cells were treated with 100 μL NB_H_. For the single VEGFR2-targeted group, SVR and 4T1 cells were treated with 100 μL NB_V_. A certain amount of DMEM were also added to the 24-well plates ensuring that the final volume per well was 500 μL. Four kinds of cells that were not treated with targeted or non-targeted PLGA NBs were used as controls. After 30 min of culture, the medium containing the NBs was removed and the cells were washed three times with PBS. Then, the cells were fixed at room temperature for 15 min with 4% paraformaldehyde, washed with PBS and stained by a nucleus staining agent (DAPI; Beyotime Biotechnology Co., Ltd, Shanghai, China). The visualization of fluorescent staining was performed using a laser scanning confocal microscopy (LSCM) (Leica TCS SP5 II, Leica Microsystems Ltd, Mannheim, Germany) to verify the targeting of the NBs onto cell surfaces. All experiments were carried out in triplicate.

Flow cytometry (FCM) was carried out to observe the conjugation rate between antibody–nanobubble bioconjugates and the cells. The groupings were consistent with the LSCM assay. SKBR3, MDA-MB-231, SVR and 4T1 cells were cultured and treated as described above. All the cells were centrifuged (1500 rpm, 5 min) after trypsin-digested and collected in test tubes prior to FCM with a density of 1 × 10^5^ cells per tube. The fluorescence intensity of the cells was measured by using a fluorescence-activated cell sorter (FACS) Calibur flow cytometer (Beckman Coulter, Fullerton, CA, USA) and the data were analyzed by Win MDI softwares. All experiments were carried out in triplicate.

### *In vitro* US imaging

A preliminary evaluation of the *in vitro* US contrast behavior of the targeted and non-targeted PLGA NBs was carried out by using ultrasonic diagnostic instrument (MyLab Twice; Esaote SpA, Genova, Italy) with a SL3116 transducer. 2 mg/mL of NB_C_, NB_V_, NB_H_ and NB_D_ in 2 mL plastic eppendorf tube were imaged in a tank filled with degassed and deionized water at 37 °C. Pure degassed and deionized water was injected in the same tube to serve as background control. The samples were scanned using the transducer in conventional B mode (mechanical index, MI = 0.06; frequency, 22 MHz; Gain = 70%; Depth = 15 mm) in order to observe the imaging ability. Strong dotted echoes in the tubes filled with NB_C_, NB_V_, NB_H_ and NB_D_ was drawn as a region of interest (ROI). The average gray -scale intensity for each ROI was computed by the image analysis software developed independently by Shanghai Jiao Tong University (DigSubAna).

### Determination of US-mediated NBs destruction

To further confirm US-mediated NBs destruction, an *in vitro* experiment was carried out using the same instruction and probe described above. 2 mg/mL of dual-targeted NBs in 2 mL plastic eppendorf tube were imaged in a tank filled with degassed and deionized water at 37 °C, and then exposed to a continuous 16-MHz high-power destruction pulse with a MI of 0.3 for 5 s. Immediately after US exposure, 10 μL of NBs solution was diluted 100 times, and 1 mL of diluted solution was analyzed. The number of remaining NBs was counted using the NanoSight NS300 (Malvern Instruments, Ltd., Malvern, UK). Before the exposure to US, 10 μL of dual-targeted NBs solution was taken out and diluted 100 times, then 1 mL of diluted dual-targeted NBs were used as a control. This experiments were carried out in triplicate.

### *In vitro* ultrasonic target-specific enhancement

SKBR3, MDA-MB-231, SVR and 4T1 cells were incubated for 30 min with medium alone or targeted NBs and they were divided into three groups as follows: the control group, the single-targeted group and the dual-targeted group. Four kinds of cells were treated as described above, which were consistent with the LSCM assay and FCM measurements. Confluent monolayers of four kinds of cells were detached with trypsin/EDTA and dissociated into cell suspensions. All the cells were centrifuged (1500 rpm, 5 min) and collected in test tubes with 1 mL sterile PBS.

Agarose gels (1%) were prepared with 20 mL dishes. 0.5 mL of cell suspension in the sample tube were extracted separately using 1 mL syringe. To simulate the targeting of the NBs onto a tissue surface (such as a vascular or cell site), different kinds of cell suspensions (with or without treatment with targeted NBs) were injected slowly to agarose gels of the dishes and immediately imaged by a Vevo 770 high-resolution US imaging system (VisualSonics Inc., Toronto, Canada). The system settings for all imaging sessions were identical: Gain = 14 dB; TGC = 0 at all locations; Central frequency = 40 MHz; MI = 0.08. B-mode images were acquired at 8 bit amplitude resolution with a pixel gray-scale range of 0–255. Three frame images were obtained from each cell suspension.

The difference of ultrasonic signal intensity in each group was qualitatively observed to evaluate the effect of target-specific *in vitro* enhancement. The bright line of the ultrasonic image was drawn as a ROI. The average gray-scale value for each ROI was also computed by the DigSubAna image analysis software.

### Tumor model

Subcutaneous tumors were established in 28 athymic female BALB/c mice in random order by subcutaneous injection of a suspension of 3 × 10^6^ SKBR3 cells in 30 μL PBS in the right flank region. Two hundred microliters of estrogen (0.3 mg/mL, Beyotime Biotechnology Co., Ltd, Shanghai, China) was intraperitoneally injected into nude mice every other day to induce breast cancer. Tumors were allowed to grow for 10–15 days. Finally, subcutaneous tumors successfully developed on 26 of 28 animals injected with SKBR3 cells, and mean maximum diameter measured at US was 5.7 ± 0.5 mm (range, 4.8–8.2 mm). Twenty tumor-bearing mice were used for the *in vivo* study of targeted contrast-enhanced US imaging, and the remaining six were used for fluorescence molecular imaging of small animal tumor models.

### *In vivo* targeted contrast-enhanced US with 22 MHz probe

The preparations of targeted and non-targeted NBs were consistent with the method used in our *in vitro* study. The final concentration of the solution was 4 mg/mL. Twenty mice bearing tumors were averagely divided into four subgroups as follows: non-targeted PLGA NBs (NB_C_), HER2-targeted PLGA NBs (NB_H_), VEGFR2-targeted PLGA NBs (NB_V_) and dual-targeted PLGA NBs (NB_D_). Tumor-bearing mice were anesthetized intraperitoneally with 4.5% chloral hydrate (0.1 mL/10 g) prior to agent injection. In the each subgroup of 20 tumor-bearing mice, 4 mg/mL of NB_C_, NB_H_, NB_V_ and NB_D_ were separately injected manually through the tail vein (NBs volume, 0.4 mL per injection; injection time, 3 s) during the same imaging session.

### Image acquisition and quantification

One radiologist (J.D., with 11 years of experience in performing US) performed real-time, two-dimensional fundamental brightness-mode (B-mode) targeted contrast-enhanced US by using ultrasonic diagnostic instrument (MyLab Twice; Esaote SpA, Genova, Italy) and a 22-MHz high-frequency linear transducer (SL3116). Gain was 70%; focal distance was 7 mm; transmit power was 10%; and MI was 0.1. Images were acquired at a 20-Hz frame rate. The US probe was positioned 2 to 3 mm above the tumor so that the central portion of the tumor was contained within the focal zone of the US transducer. The probe position, gain settings and midfield focus were initially optimized and maintained throughout each experiment. To decrease speckle variance, both the US probe and the animal were fixed and remained at the same position throughout the study.

The goal of the ultrasonographic image acquisition and analysis protocol was to differentiate the backscattered acoustic signal due to NBs retained by the tumor from the background signal of the tumor itself and NBs still freely circulating in the bloodstream. For this purpose, we used previously described principles of US-induced microbubble destruction and replenishment. In our study, the method of image acquisition and analysis was consistent with those used in the previous studies^4,17,20^. In their studies, a continuous 10-MHz high-power destruction pulse was applied (a MI of 0.235) for 3 s to destroy all the microbubbles within the beam elevation. The frequency of the probes (16-MHz) used in our study was relatively higher, which led to the oscillations of NBs were not as well under the low frequency ultrasound. In addition, our NBs were prepared by the polymeric shell with higher compression resistance. Therefore, in our study, a higher mechanical index (a MI of 0.3) and a longer time (5 s) were used for destroying the NBs.

Imaging was suspended for 6 min after injection of NBs. This time allowed binding and retention of targeted NBs while awaiting wash-out of the unbound contrast agent. After the 6-minute waiting period, 120 imaging frames of the tumor were acquired during 6 s. A continuous (16-MHz) high-power US destruction sequence was then applied (a MI of 0.3) for 5 s, which destroyed the NBs within the beam elevation. After the destruction pulse, the system was reset with identical imaging parameters as before the destruction event. Following destruction (10 s were given to allow freely circulating NBs to refill into tumor vessels), another set of images (120 frames) was then acquired during 6 s. In the same 20 tumor bearing mice, targeted US of normal skeletal muscle (hind limb adductor muscles as a quasi tumor negative model) was performed as described previously for tumor imaging to assess contrast enhancement of nonneoplastic and nonangiogenic microvasculature after injection of NB_V_, NB_H_ and NB_D_.

Image processing and quantification were also performed with the software developed independently by Shanghai Jiao Tong University (DigSubAna). Two sets of images (a predestruction set and a postdestruction data set as a reference) were used for image processing. The average video intensity of predestruction and postdestruction (background) sonograms was measured in a ROI encompassing the examined tumor. The difference in video intensity between predestruction and postdestruction ultrasonographic frames corresponded to the imaging signal attributable to targeted UCA adherent to molecular endothelial and cell biomarkers. The subtracted image was generated and displayed as a color (green) overlay on the B-mode image by the software to provide a map of the spatial distribution of the UCA retained by the tissue. For assessment of contrast enhancement in hind limb muscles, a ROI was set to encompass the adductor muscle.

### *In vivo* targeted US imaging with 50 MHz probe

In order to further demonstrate the enhancement of US images from the bonded and retentive NBs within tumors and the accuracy of the 6-minute waiting period before the US destruction, another different type of *in vivo* US imaging system using 50 MHz probe was adopted to investigate the targeted contrast-enhanced capability of dual-targeted NBs in breast tumor mice model.

The imaging procedure was conducted using a dedicated small-animal high-resolution ultrasound imaging system (Prospect 3.0, S-Sharp Corporation, Taiwan) and a PB406e (frequency range: 30~50 MHz) high-frequency linear transducer with following parameters: frequency, 50-MHz; dynamic range, 50 dB; Depth, 3.7 mm; Power, 35%. Ten breast tumor-bearing mice were averagely divided into two subgroups as follows: non-targeted NBs and dual-targeted NBs. Animals were kept on a heated stage to maintain their body temperature. US gel was used as a coupling agent on the tumor of the mice. The acoustic focus was placed at the center of the tumor at the level of the largest plane. Then, the imaging mode was converted to contrast mode and 1000 imaging frames were collected during a period of 8 min after the administration of dual-targeted NBs and non-targeted NBs (concentration: 4 mg/mL; in a volume, 0.4 mL per injection). The US images were recorded digitally by overlaying the US enhanced signals (green color) on the background and the gray-scale intensity-over-time curves were obtained in ROI encompassing the whole tumor in the imaging plane.

### Immunofluorescence and immunohistochemical staining of tumors

Animals were euthanized after US, and the subcutaneous tumors were excised, embedded in optimal cutting temperature compound, and frozen on dry ice. Frozen blocks were sectioned at 5 μm and mounted on glass slides for immunofluorescence and immunohistochemical staining. A double-staining procedure was employed to visualize VEGFR2 expression on tumor endothelial cells. The following method below was used for mouse VEGFR2 staining: The slices were incubated with rat anti-mouse VEGFR2 primary antibody with dilution of 1:100 (Google Biotechnology Co., Ltd, Wuhan, China) overnight at 4 °C and visualized by using Cy3-conjugated goat anti-rat secondary antibody in a 1:300 ratio (Google Biotechnology Co., Ltd, Wuhan, China). Consecutive slices from each sample were used for CD31 staining (a marker for endothelial cells). For this purpose, the slices were incubated with a goat anti-mouse CD31 antibody in a 1:50 ratio (Google Biotechnology Co., Ltd, Wuhan, China) at room temperature for 1 hour and visualized with FITC-conjugated donkey anti-goat antibody in a 1:200 ratio (Google Biotechnology Co., Ltd, Wuhan, China). The slides were placed in PBS (PH7.4) of decolorization shaking table for 5 min. After drying, DAPI dye was added to the slides, and samples were protected from light and incubated for 10 min. Fluorescent images were acquired with an inverted fluorescence microscope (Nikon Eclipse Ti-SR, Nikon, Tokyo, Japan). The expression of HER2 in SKBR3 breast cancer was detected by immunohistochemical staining. The slides were stained for human HER2 by using a rabbit anti-human primary antibody, with dilution of 1:200 (Google Biotechnology Co., Ltd, Wuhan, China); a HRP-conjugated goat anti-rabbit secondary antibody, with dilution of 1:200 (DAKO, Hamburg, Germany). Positive staining was localized to plasma membrane.

### Small animal fluorescence imaging

To further confirm binding specificity of dual-targeted PLGA NBs to both VEGFR2 and HER2, *in vivo* small animal fluorescence imaging was performed in another six tumor-bearing mice. Six tumor-bearing mice were averagely divided into two groups: non-targeted PLGA NBs (NB_C_ group) and dual-targeted PLGA NBs (NB_D_ group). Tumor-bearing mice were anesthetized intraperitoneally with 4.5% chloral hydrate (0.1 mL/10 g) prior to agent injection. The NB_D_ were suspended in deionized water at a concentration of 4 mg/mL and 0.4 mL solution was administered via the tail vein injection. The NB_C_ solution as a control, was administered via the tail vein injection at the same concentration and volume. At 5, 15, 30, 45 and 60 min post-injection, the fluorescence images were captured with a 1-s exposure time using the small animal *in vivo* fluorescence imaging system (LB 981, Berthold Technologies Gmbh & Co. KG, Germany).

### *In vivo* toxicity analysis of nude mice

Twelve nude mice were chosen as animal model to study *in vivo* toxicity of dual-targeted NBs, which was intravenously injected into the mice via tail vein with a concentration of 4 mg/mL and a volume of 0.4 mL. At 4 h, 12 h, 24 h and 72 h post-injection of dual-targeted NBs, three nude mice were sacrificed separately, and tissues (heart, liver, spleen, lung and kidney) along with blood samples were collected for further analysis. Another three mice receiving the injection of only PBS (0.4 mL per injection) were chosen as the control group. Serum biochemistry tests were performed for quantitative evaluation about two important hepatic indicators (alanine aminotransferase (ALT), aspartate aminotransferase (AST), and one indicator for cardiac function (creatinine kinase MB isoenzyme (CK-MB)) and two indicators for kidney functions (creatinine and blood urea nitrogen (BUN)). Tissues (heart, liver, spleen, lung and kidney) were fixed in 10% formaldehyde solution, embedded in paraffin, and cut into 5-μm-thick sections. Subsequently, the sections were stained with hematoxylin and eosin (HE) to observe the histopathological abnormalities or tissue damage using microscopy (ZEISS, Axioplan 2 Imaging, Jena, Germany). All the specimens were observed by two investigators who were blinded to the experiment and received no information about the specimens.

### Statistical analysis

Quantitative data were expressed as the means and standard deviation (mean ± SD). Data from two independent samples were analyzed with Student’s t test. Analysis of variance (ANOVA) was used to determine the significance of differences in multiple comparisons. A *P* value of less than 0.05 was considered statistically significant. Statistical analyses were performed using SPSS software version 13.0 (SPSS Inc; Chicago, IL, USA).

### Data availability statement

All data used for this article are publicly available.

## Results

### Characterization of NBs

**Figure 1:**
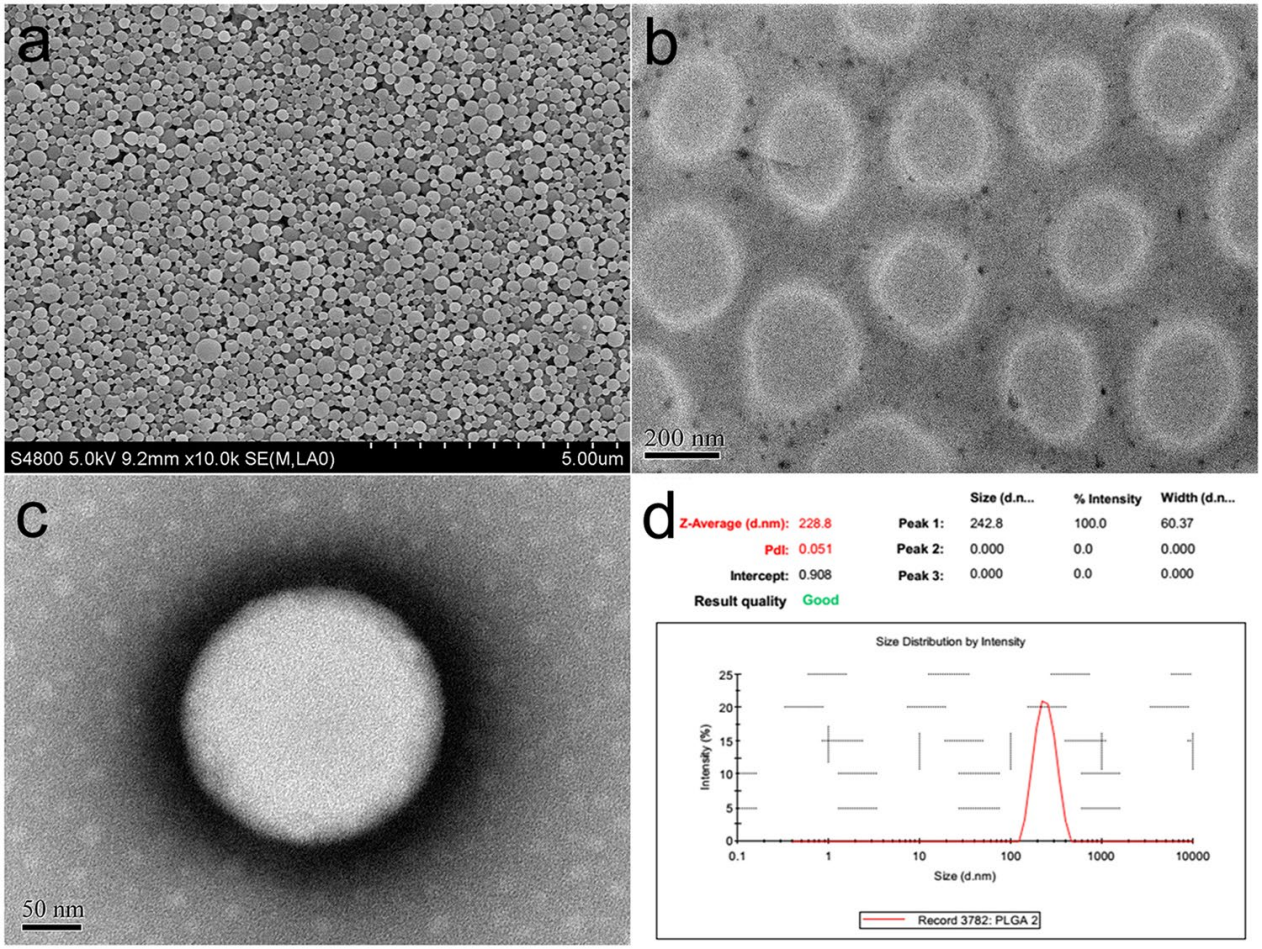
Characterization of C_3_F_8_-filled PLGA NBs. (**a**) SEM photograph of C_3_F_8_-filled PLGA NBs. (**b**) TEM image of the synthesized hollow PLGA NBs. (**c**) TEM image of C_3_F_8_-filled PLGA NBs strained negatively with phosphotungstic acid. The typical capsular structure is visualized, where the polymeric shell appears darker. (**d**) Average size and distribution of C_3_F_8_-filled PLGA NBs measured by dynamic laser scattering.

Hollow polymeric NBs were prepared from PLGA polymer containing free carboxylic end groups, using an adapted oil-in-water emulsion solvent evaporation process. SEM photograph confirmed that the C_3_F_8_-filled PLGA NBs had a regular spherical morphology (Fig. 1a). The TEM images revealed the capsular structure of the C_3_F_8_-filled PLGA NBs making by the camphor sublimation (Fig. 1b). The typical core-shell structure of the C_3_F_8_-filled PLGA NBs strained negatively with phosphotungstic acid was more clearly visualized, with the PLGA-based shell exhibiting a darker contrast than the background (Fig. 1c). Based on the TEM images, the thickness of the shell was found to be 35 nm. As indicated in Fig. 1d, the mean diameter of the NBs prepared in the present study was 228.8 ± 60.4 nm and the polydispersity index was 0.051, revealing uniform size and good dispersion of the NBs. The zeta potential of the NBs was −24.7 ± 5.1 mV. The overall negative charge of C_3_F_8_-filled PLGA NBs could guarantee the stability of the NBs in water. There were no obvious changes in the sizes between NB_C_ and the single- or dual-targeted NBs. The mean diameter of the dual-targeted NBs was 230.2 ± 58.5 nm and the zeta potential was −18.4 ± 4.7 mV.

### *In vitro* targeting ability

**Figure 2:**
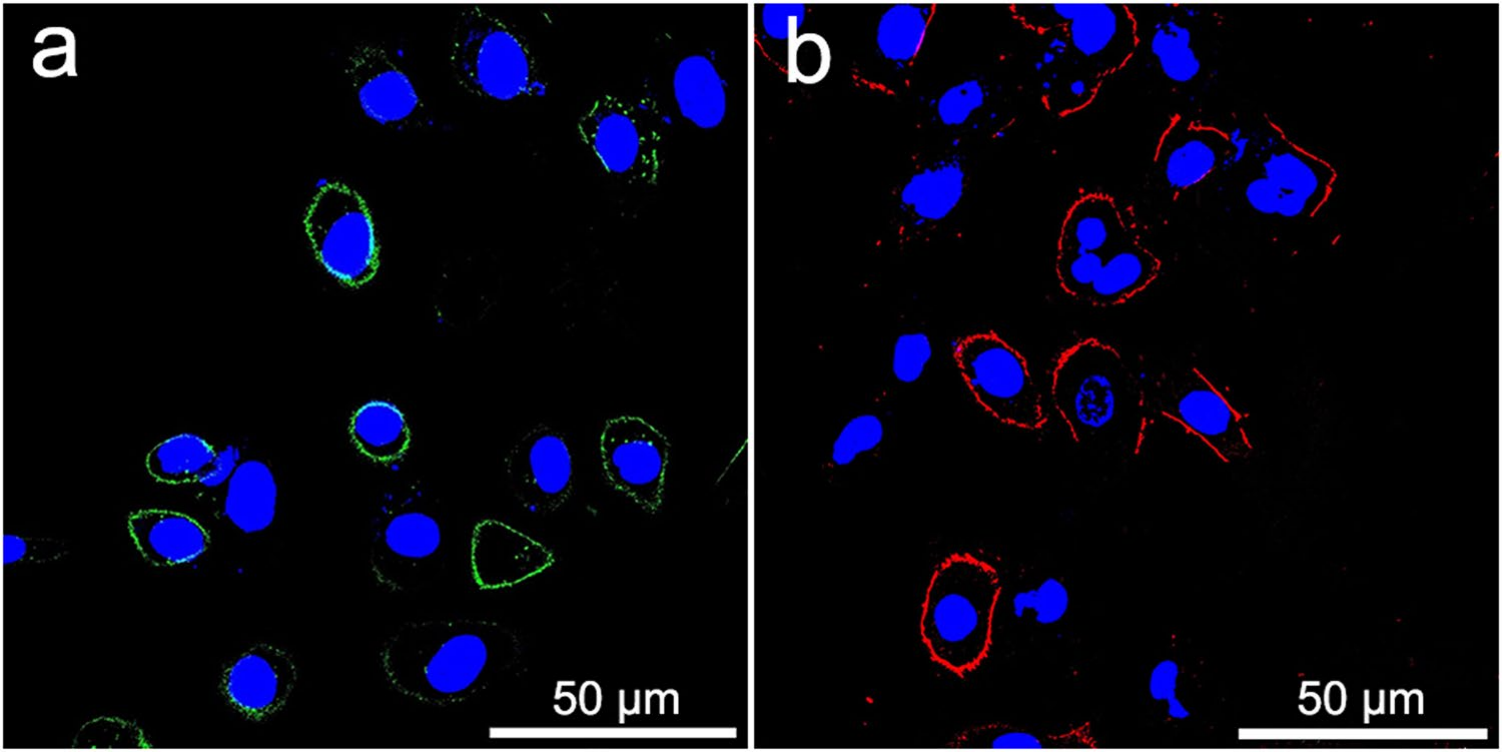
Confocal microscope images of SKBR3 and SVR cells treated with single-targeted NB_H_ and NB_V_. (**a**) NB_H_ + SKBR3. (**b**). NB_V_ + SVR. Green, fluorescence signal of anti-HER2 antibody labeled with FITC. Red, fluorescence signal of anti-VEGFR2 antibody labeled with PE. Blue, nuclei stained with DAPI.

**Figure 3:**
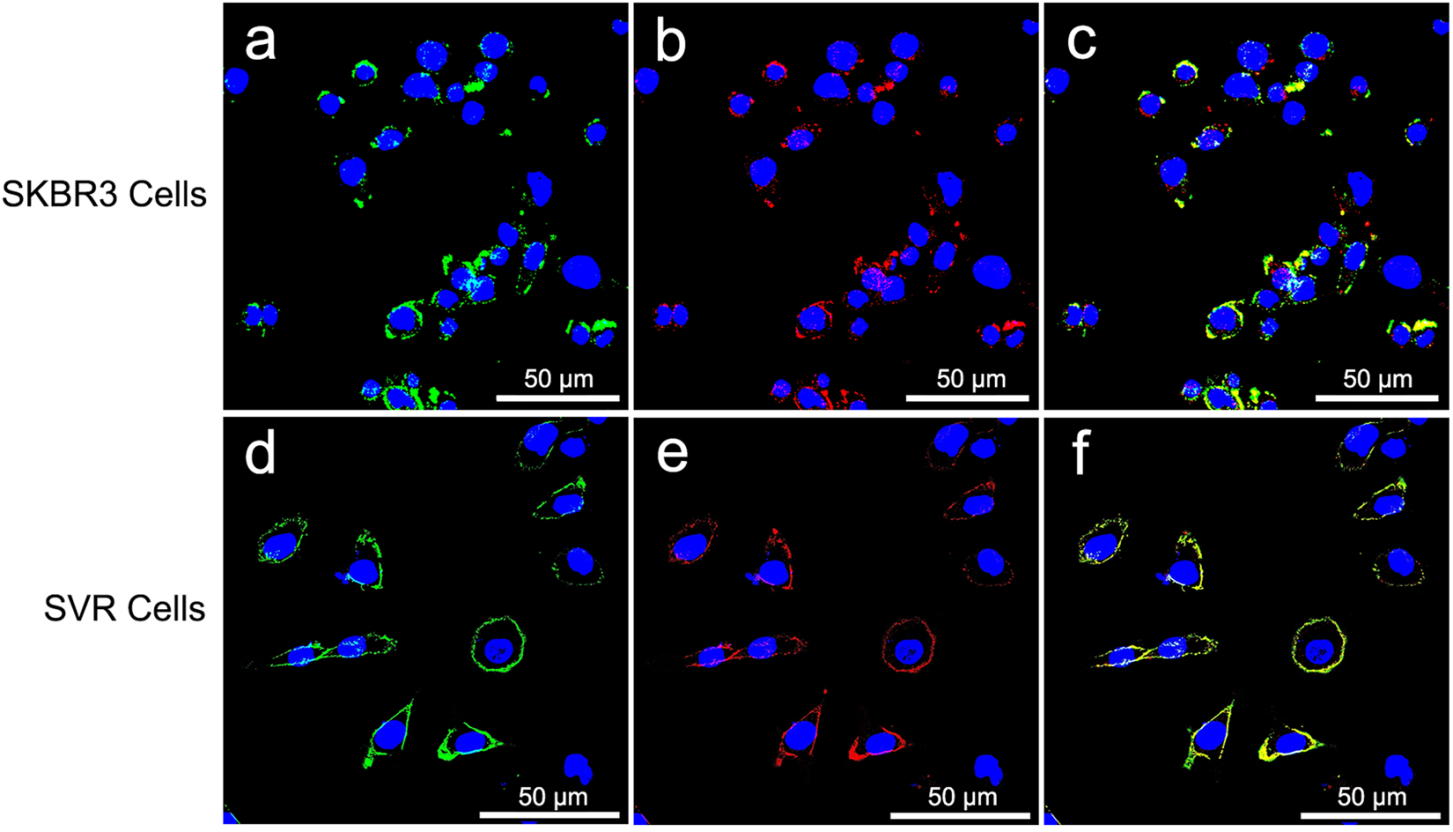
Confocal microscope images of SKBR3 and SVR cells incubated with dual-targeted NB_D_. (**a**–**c**) SKBR3 cells were substantively stained by nanoparticle-antibody bioconjugates (green, red or yellow, incomplete circles) around cell nuclei. (**d**–**f**) SVR cells were strongly stained by nanoparticle-antibody bioconjugates (bright, complete green, red or yellow circles). Green, fluorescence signal of anti-HER2 antibody labeled with FITC. Red, fluorescence signal of anti-VEGFR2 antibody labeled with PE. Yellow, merged fluorescence of red and green. Blue, nuclei stained with DAPI.

We utilized LSCM to confirm the *in vitro* targeting ability of single- or dual-targeted PLGA NBs. Only green or red fluorescence was found on the membranes of SKBR3 cells or SVR cells after the incubation with NB_H_ or NB_V_ (Fig. 2). The NB_D_ generated substantial membrane staining of SKBR3 cells (Fig. 3a–c) and SVR cells (Fig. 3d–f). Both green and red fluorescence could be detected on the membrane of the same cell, and the overlap of red and green fluorescence gave a yellow color, demonstrating the targeting attachments and the receptor-mediated specificity of dual-targeted PLGA NBs. On the contrary, negligible fluorescence was detected on the membranes of SKBR3 or SVR cells treated with the non-targeted PLGA NBs. In addition, little fluorescence was observed when MDA-MB-231 and 4T1 cells were incubated with single- or dual-targeted PLGA NBs, confirming the lack of HER2 and VEGFR2 expressions on their membranes. Therefore, the qualitative targeting behavior of the single- or dual-targeted PLGA NBs was visually verified by LSCM images.

### Specific binding rate detected by FCM

**Figure 4:**
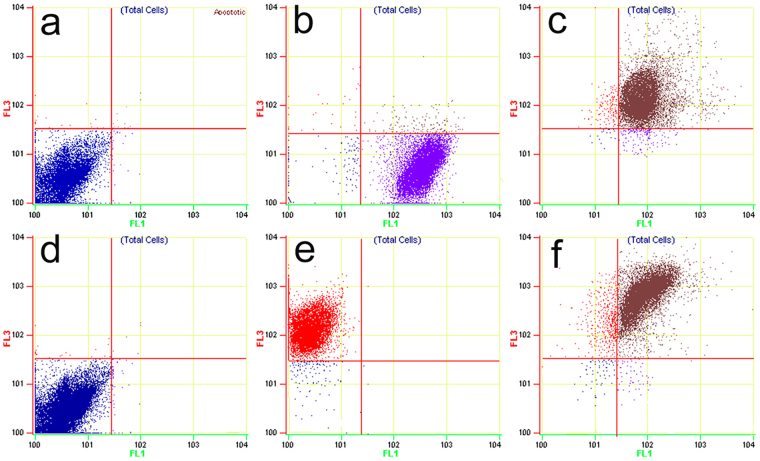
FCM measurements of the binding efficiencies of targeted PLGA NBs to corresponding target and non-target cells. (**a**) NB_D_ + MDA-MB-231 cells. (**b**) NB_H_ + SKBR3 cells. (**c**) NB_D_ + SKBR3 cells. (**d**) NB_D_ + 4T1 cells. (**e**) NB_V_ + SVR cells. (**f**) NB_D_ + SVR cells. FLI channel shows FITC signal and FL3 channel shows PE signal.

The specific binding rate of the targeted PLGA NBs conjugating toward cells was validated quantitatively via FCM, and the binding efficiencies of targeted PLGA NBs and corresponding target and non-target cells were shown in Fig. 4. As shown in Table 1, the binding rates of single-targeted and dual-targeted PLGA NBs to SKBR3 or SVR cells were both more than 70%. Although NB_D_ had higher binding rates with SKBR3 and SVR cells than single-targeted NBs, no significant difference was found between them (*P* = 0.098; *P* = 0.085, respectively). MDA-MB-231 (HER2 negative) cells and 4T1 (VEGFR2 negative) cells were not recognized by nanoparticle-antibody bioconjugates, suggesting that the targeted NBs had minimal non-specific attachment to cells. The binding rates of both the single-targeted and the dual-targeted NBs to SKBR3 cells were significantly higher than those of the targeted NBs to MDA-MB-231 cells (*P* < 0.001; *P* < 0.001, respectively). Similar results was also observed in the study for the binding affinity of the targeted NBs to VEGFR2-overexpressing SVR and negative control 4T1 cells. Non-targeted NB_C_ had no obvious target binding to the four kinds of cells. FCM results provided further strong evidence that targeted NBs had the specific binding ability of recognizing and conjugating with the SKBR3 and SVR cells, which were known in overexpressing HER2 and VEGFR2.

**Table 1:** The binding rates to target and non-target cells of the different groups.

Cells	Non-targeted NBs	Single-targeted NBs	Dual-targeted NBs
SKBR3	2.17 ± 0.56	70.16 ± 3.28*^‡^	74.59 ± 3.47*^‡^
SVR	2.35 ± 0.66	73.49 ± 3.67*^‡^	78.41 ± 3.41*^‡^
MDA-MB-231	1.58 ± 0.24	2.28 ± 0.72	2.37 ± 1.21
4T1	1.81 ± 0.39	2.74 ± 1.03	3.24 ± 1.16

Note—data in table are percentages.

^*^Significantly different from the cells treated with non-targeted NBs (*P < 0.001).

^‡^Significantly different from MDA-MB-231 or 4T1 cells treated with single or dual-targeted NBs (^‡^*P* < 0.001).

### *In vitro* US imaging

**Figure 5:**
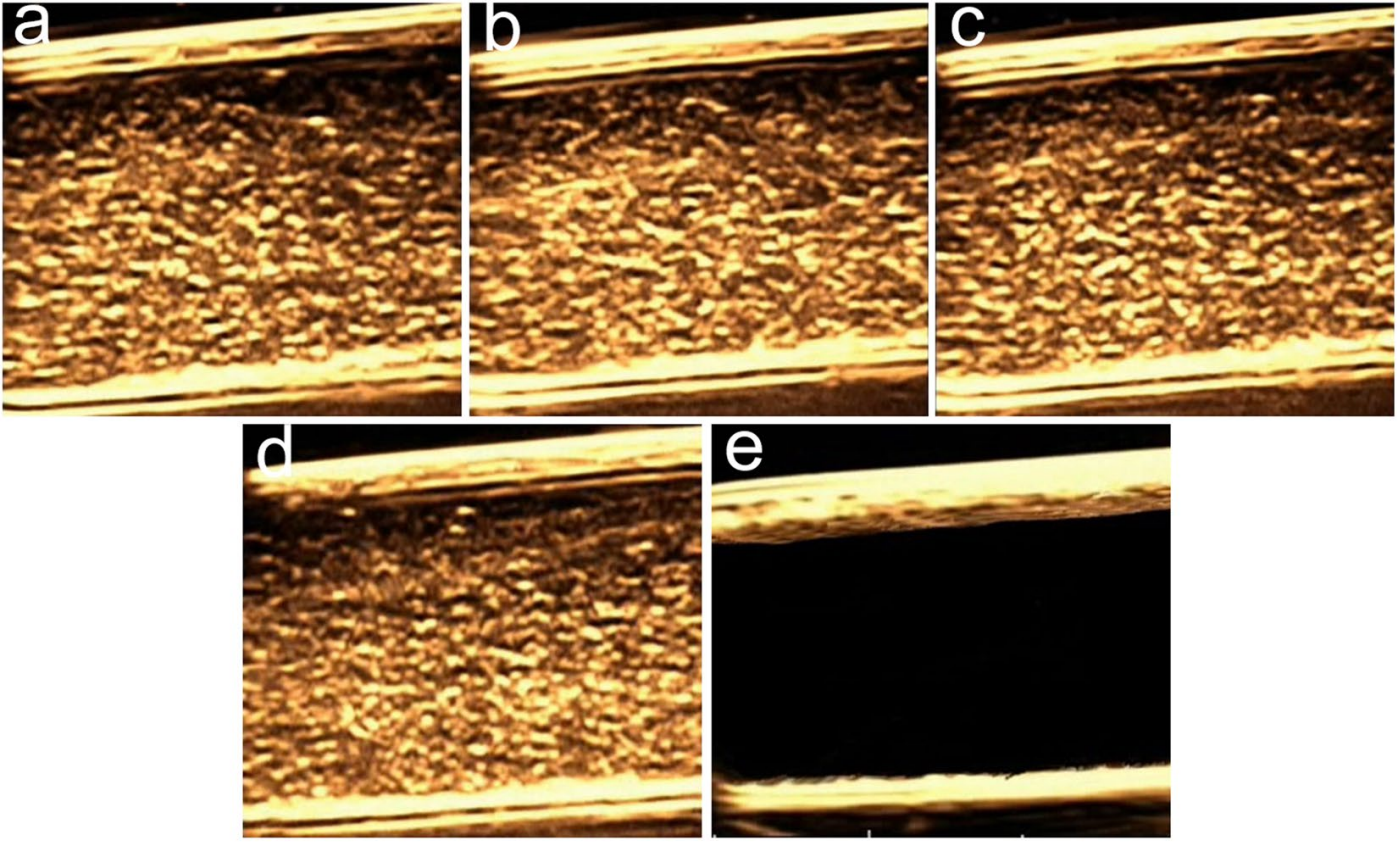
*In vitro* US imaging effects of the same concentration (2 mg/mL) of (**a**) NB_C_, (**b**) NB_H_, (**c**) NB_V_, (**d**) NB_D_ and (**e**) degassed water at 22 MHz.

The capability of targeted PLGA NBs as a contrast agent for US imaging was assessed *in vitro* using the B-scan imaging mode at a frequency of 22 MHz and at a MI of 0.06. As shown in Fig. 5, the tubes filled with the same concentrations (2 mg/mL) of the NB_C_, NB_H_, NB_V_ and NB_D_ displayed strong dotted echoes in B-mode images and produced good imaging effects (NB_C_ = 120.0 dB, NB_H_ = 123.2 dB, NB_V_ = 121.2 dB and NB_D_ = 120.7 dB, respectively), there was almost no visible difference between the different groups. The echo signal of targeted PLGA NBs were similar to that of non-targeted PLGA NBs, whereas the tube filled with degassed water was observed as anecho (0.78 dB). In addition, the length of time during which the signal enhancement was produced and sustained by 2 mg/mL of NB_D_ at 22 MHz was investigated. The signal intensity of 2 mg/mL NB_D_ remained strong until 1 min, there was slight decrease at 2 min and signal enhancement could still be detected at 3 min. It was notable that the slight signal enhancement could also be observed even at 4 min. These *in vitro* results demonstrated that the NB_D_ were able to be used as a contrast agent for efficient US imaging, and the length of imaging time could meet the requirement of clinical contrast-enhanced US.

### Evaluation of NBs destruction by counting

When a continuous 16-MHz high-power destruction pulse was applied (a MI of 0.3) for 5 s, the remaining NBs significantly decreased from 3.16e + 008 ± 2.67e + 006 to 4.94e + 007 ± 8.54e + 006 (*P* < 0.001) and 84.37% of NBs was destroyed within the beam elevation. This result obtained from *in vitro* experiment would aid in determining US exposure intensity for *in vivo* small animal tumor models.

### *In vitro* ultrasonic target-specific enhancement

**Figure 6:**
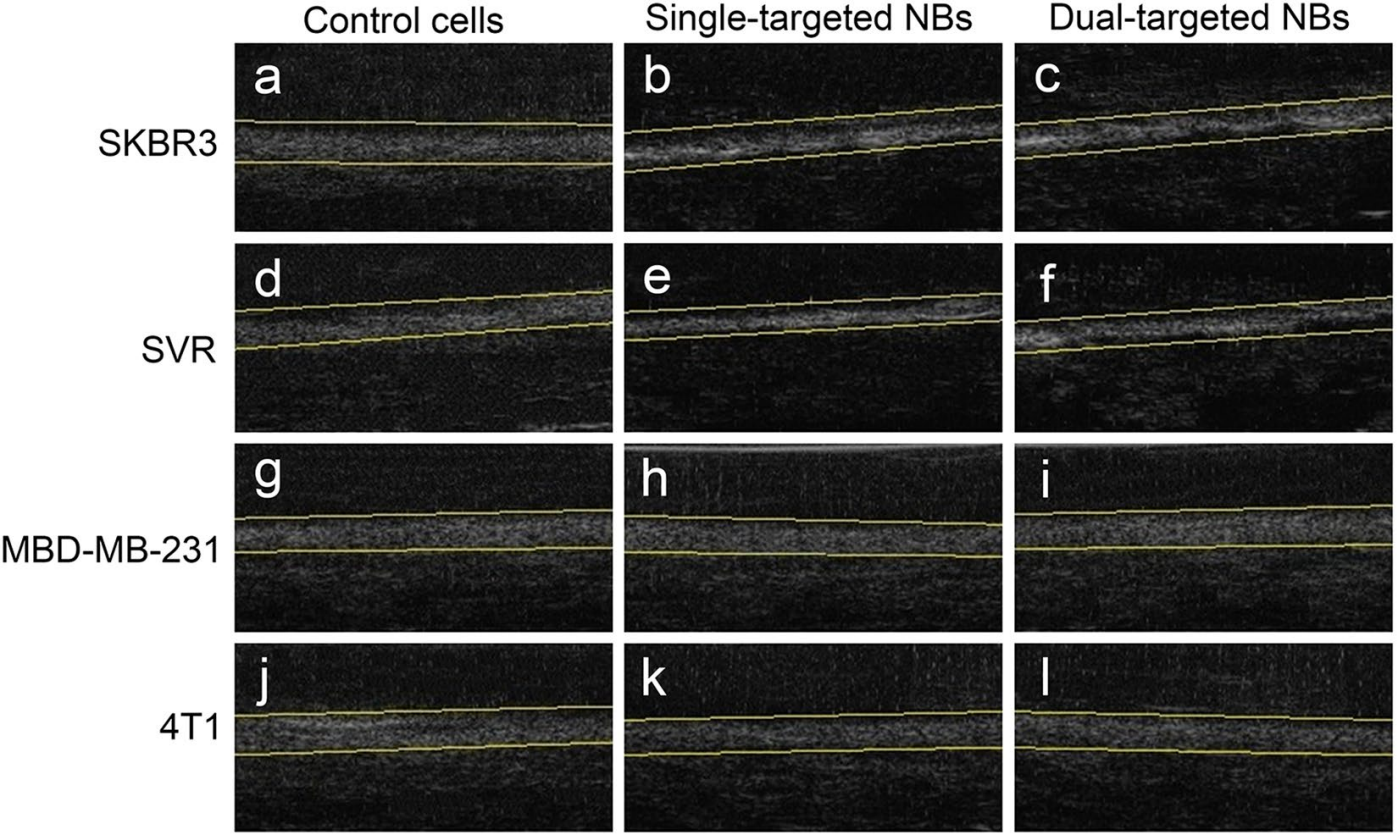
*In vitro* US imaging effects of different cells with and without targeted NBs treatment at 40 MHz. (**a**) SKBR3 cells, (**b**) NB_H_ + SKBR3 cells, (**c**) NB_D_ + SKBR3 cells, (**d**) SVR cells, (**e**) NB_V_ + SVR cells, (**f**) NB_D_ + SVR cells, (**g**) MDA-MB-231 cells, (**h**) NB_H_ + MDA-MB-231 cells, (**i**) NB_D_ + MDA-MB-231 cells, (**j**) 4T1 cells, (**k**) NB_V_ + 4T1 cells, (**l**) NB_D_ + 4T1 cells.

Both the cells treated with targeted NBs and simple control cells shown in the gel displayed the bands of exquisite high echoes with uniform distribution and clear boundary. As shown in Fig. 6, the echo bands of SKBR3 and SVR cells treated with single- or dual-targeted PLGA NBs appeared brighter and more hyperdense in US B-mode images compared to those of the original cells without targeted NBs treatment. However, the change in echogenicity was not found in MDA-MB-231 and 4T1 cells treated with the targeted NBs.

Four kinds of the cell suspensions were imaged as well, and the brightness of the images was quantified by the average gray-scale value of the ROIs defined on the B-mode images. The ultrasonic gray-scale values of the different experimental groups were shown in Table 2. The average gray-scale values for SKBR3 and SVR cells treated with single- or dual-targeted NBs were significantly higher than those of the control SKBR3 and SVR cells (*P* < 0.05 for all comparisons). However, there were no significant differences in the average gray-scale values for SKBR3 and SVR cells between the dual-targeted and single-targeted groups (*P* > 0.05 for all comparisons). The average gray-scale values between SKBR3 and MDA-MB-231 cells or SVR and 4T1 cells were significantly different in both the single-targeted and dual-targeted groups (*P* < 0.05 for all comparisons). There were no statistical differences in the average gray-scale values for MDA-MB-231 and 4T1 cells between the targeted NBs treated and control groups (*P* > 0.05 for all comparisons). Our results showed that the single- or dual-targeted PLGA NBs could specially bind to corresponding target cells *in vitro* and had better effects of target enhancement under a frequency of 40 MHz.

**Table 2:** Comparisons of the ultrasonic gray-scale values in different experimental groups (dB).

Cells	Simple cells	Single-targeted NBs	Dual-targeted NBs
SKBR3	52.33 ± 3.01	62.20 ± 3.44*^†^	65.07 ± 3.23^∗†^
SVR	53.20 ± 3.75	63.03 ± 3.16*^†^	60.73 ± 3.36*^†^
MDA-MB-231	56.80 ± 3.67	54.10 ± 3.45	57.13 ± 3.58
4T1	55.53 ± 3.74	52.83 ± 3.49	53.07 ± 3.14

**Significantly different from simple cells (*P < 0.05, ^∗^*P* < 0.01).

^†^Significantly different from MDA-MB-231 or 4T1 cells treated with single or dual-targeted NBs (^†^*P* < 0.05).

### *In vivo* targeted contrast-enhanced US with 22 MHz probe

**Figure 7:**
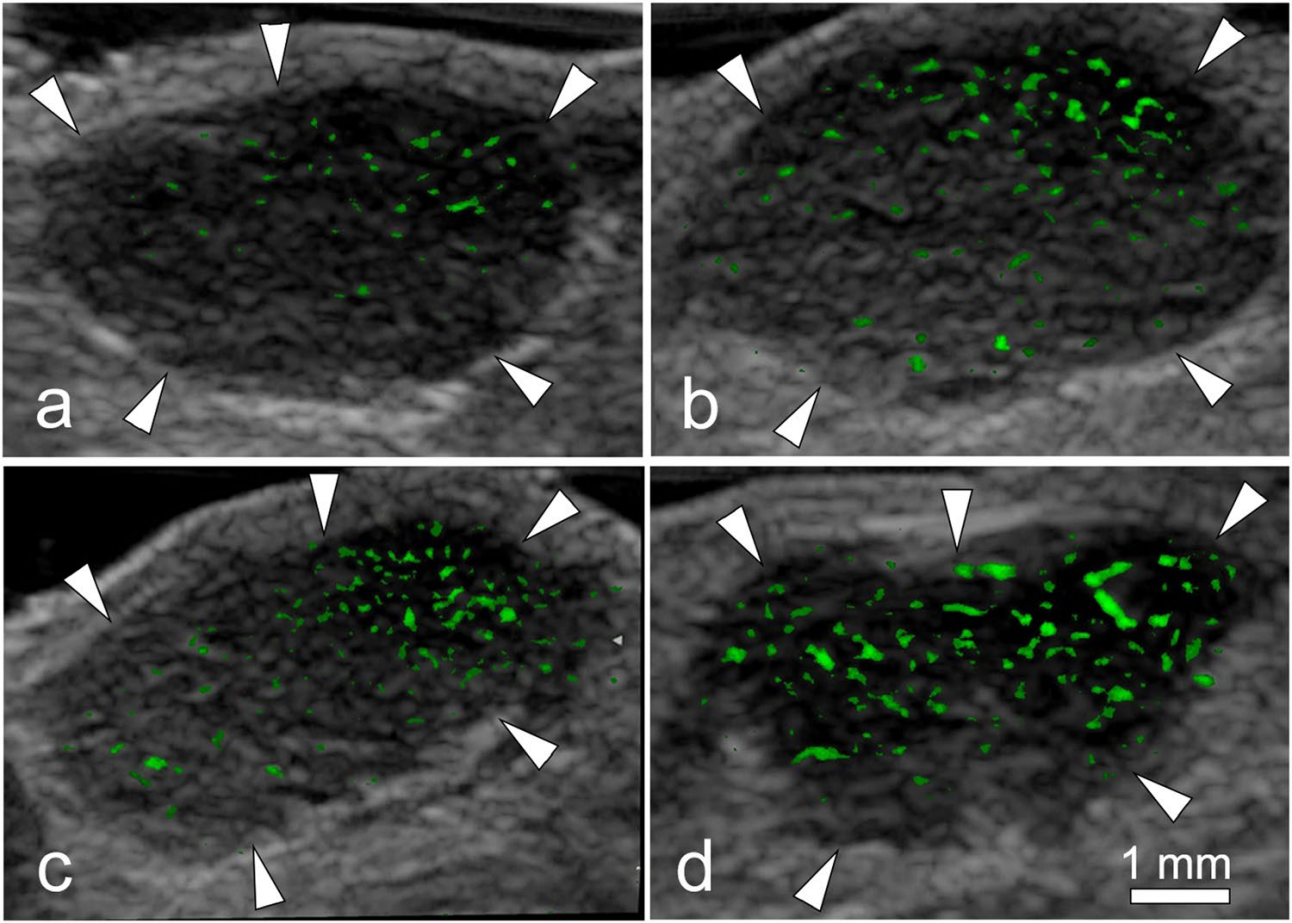
Color-coded US images in subcutaneous human breast cancer (SKBR3) xenograft tumor (arrowheads) from nude mouse. Imaging was obtained in same imaging session 6 min after intravenous injection of (**a**) NB_C_, (**b**) NB_V_, (**c**) NB_H_ or (**d**) NB_D_. Difference in video intensity from subtraction of pre- and postdestruction images (color-coded as green signal green) on gray-scale images was highest after administration of NB_D_ in the tumor.

VEGFR2-specific or HER2-specific imaging signals were measured in all tumors by using contrast-enhanced US after administration of targeted PLGA NBs. The subtracted color-coded ultrasonic image *in vivo* was shown in Fig. 7. On average, the mean difference in video intensity was 118.67 ± 4.04 dB after administration of NB_V_ and 115.33 ± 4.51 dB after administration of NB_H_ in the tumor-bearing mice. The mean difference in video intensity after administration of NB_V_ and NB_H_ showed no significant difference (*P* = 0.397). In the same imaging session, the difference in video intensity significantly increased (137.20 ± 5.30 dB versus 118.67 ± 4.04 dB, *P* = 0.001; 137.20 ± 5.30 dB versus 115.33 ± 4.51 dB, *P* < 0.001) after administration of NB_D_. It was shown that NB_D_ had a better ability of target enhancement in the tumor area, and the molecular imaging effect was better than NB_V_ and NB_H_. To confirm binding specificity of targeted PLGA NBs, NB_C_ were administered in the tumor-bearing mice, the mean difference in video intensity was 98.23 ± 4.29 dB, which was significantly decreased (*P* < 0.01 for all comparisons) compared with the mean difference in video intensity after administration of NB_V_, NB_H_ or NB_D_. Finally, as a quasi tumor–negative model, normal skeletal muscle tissue was imaged after administration of NB_V_, NB_H_ and NB_D_. No obvious color-coded signal was found in the subtraction image of hind limb adductor muscles. For all three types of targeted NBs, mean differences in video intensity measured over skeletal muscle tissue (NB_V_, 1.44 ± 0.41 dB; NB_H_, 1.38 ± 0.34 dB; NB_D_, 1.51 ± 0.36 dB) were significantly lower (*P* < 0.001 for all comparisons) compared with those measured over tumor tissue.

### *In vivo* targeted US imaging with 50 MHz probe

**Figure 8:**
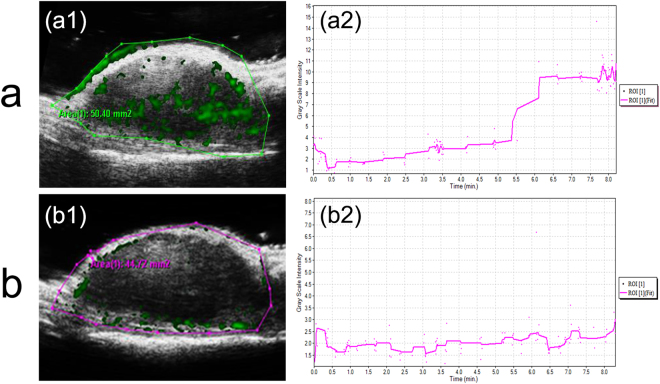
*In vivo* targeted US imaging using a 50 MHz probe for SKBR3 breast tumor-bearing mice after intravenous injections of dual-targeted NBs and non-targeted NBs. (a1 and b1) Contrast-enhanced US images for breast cancer xenograft tumor. (a2 and b2) Typical gray-scale intensity-over-time curves recorded in the corresponding tumor regions.

Compared to the mouse treated with non-targeted NBs, the significant contrast enhancement of tumor site was clearly observed (Fig. 8a1) after dual-targeted NBs had been injected into the mice via tail vein, suggesting the high *in vivo* targeted contrast-enhanced efficiency of dual-targeted NBs. In contrast to the high US signal enhancement by dual-targeted NBs, only slight contrast enhancement in the tumor was detected when non-targeted NBs was used (Fig. 8b1). Moreover, the gray-scale intensity-over-time curves starting immediately after the administrations of dual-targeted NBs and non-targeted NBs were also presented in Fig. 8(a2 and b2). In the targeted group, the signal intensity in the tumor region had a significant rise about 6 min after administration of dual-targeted NBs, and then maintained at a steady level, which might be attributed to the bonded and retentive NBs within tumors. However, in the non-targeted group, the signal intensity in the tumor region maintained at a steady and consistent low level. These positive results of *in vivo* US imaging suggested that dual-targeted PLGA NBs could be used as a targeted UCA for real-time monitoring of breast tumors.

### Small animal fluorescence imaging

**Figure 9:**
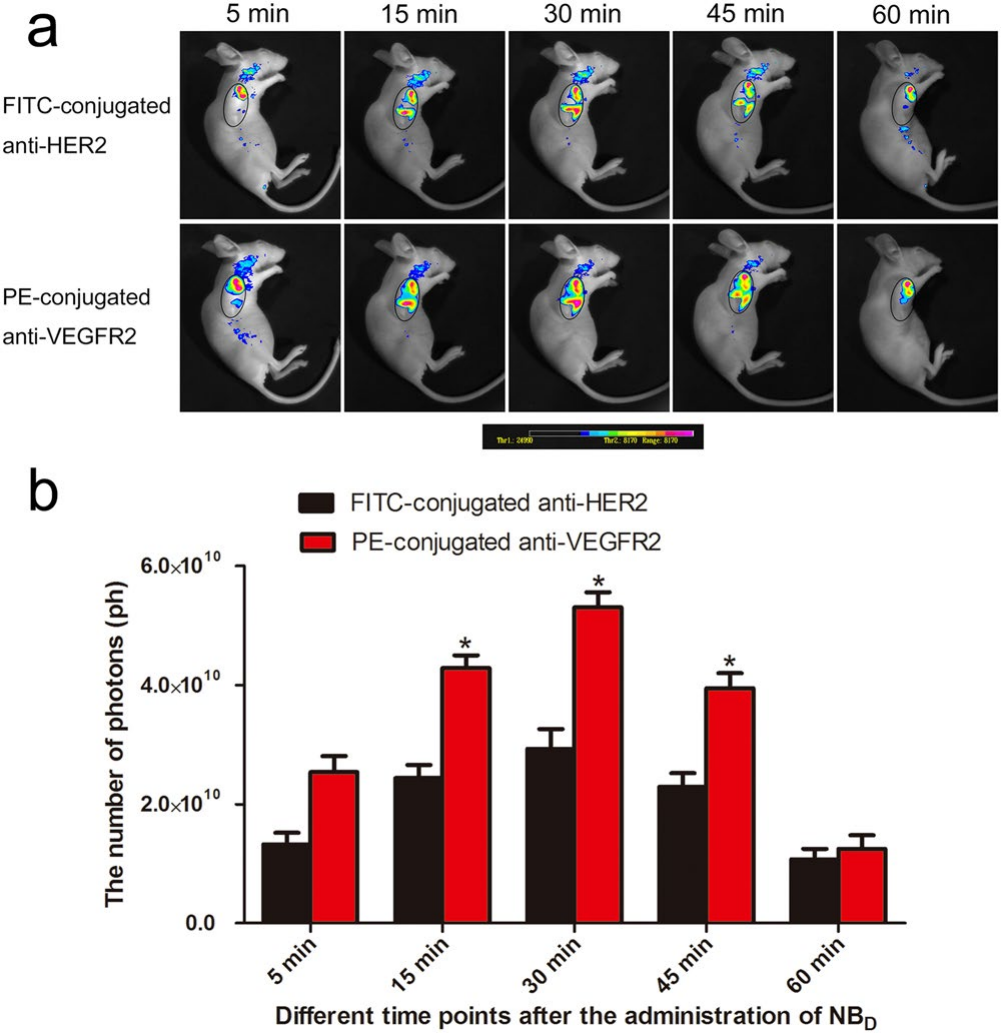
(**a**) *In vivo* time-dependent fluorescence images in breast tumor bearing-mice at different time points after the administration of NB_D_ under the different detection modes of fluorescent-conjugated antibodies. The tumor area was outlined in an oval shape. The color bar (from blue to red) indicates the change in fluorescence signal intensity from low to high. (**b**) Quantification of fluorescence signals of the tumor area at different time points after the administration of NB_D_. *P < 0.05.

*In vivo* special affinity of dual-targeted PLGA NBs to both VEGFR2 and HER2 was further evaluated using a fluorescence imaging system of small animals. At 5, 15, 30, 45 and 60 min post-injection of dual-targeted NBs, the fluorescence signal intensities of the FITC-conjugated anti-HER2 antibody and the PE-conjugated anti-VEGFR2 antibody were observed. The fluorescence imaging of tumors was presented in Fig. 9a, the control image was obtained from the mouse treated with NB_C_. As time went on, the tumor fluorescence signal of mice injected with dual-targeted NBs gradually increased, peaked at 30 min. Then, fluorescence signal of the dual-targeted NBs gradually reduced and still had not completely disappeared until 60 min. At the different postinjection time points, the PE-conjugated anti-VEGFR2 antibody of the dual-targeted NBs showed relatively stronger and larger range of fluorescence signals compared to the FITC-conjugated anti-HER2 antibody, indicating that the dual-targeted acoustic contrast agent was more easily to targetedly accumulate on the surface of tumor vessel endothelial cells. The fluorescence signal data for the tumor area also further quantitatively compared targeting efficiency of dual-targeted NBs to VEGFR2 and HER2 in SKBR3 tumors (Fig. 9b). At 15 min, 30 min and 45 min post-injection of dual-targeted NBs, the number of photons of the PE-conjugated anti-VEGFR2 antibody of dual-targeted NBs were significantly higher than those of FITC-conjugated anti-HER2 antibody (*P* = 0.026; *P* = 0.029; *P* = 0.039, respectively). In the control group, there was no significant fluorescence imaging in the tumor site. Small animal fluorescence imaging confirmed the special affinity of the dual-targeted nanosized contrast agent to tumor tissues.

### Immunofluorescence and immunohistochemistry staining of tumor

**Figure 10:**
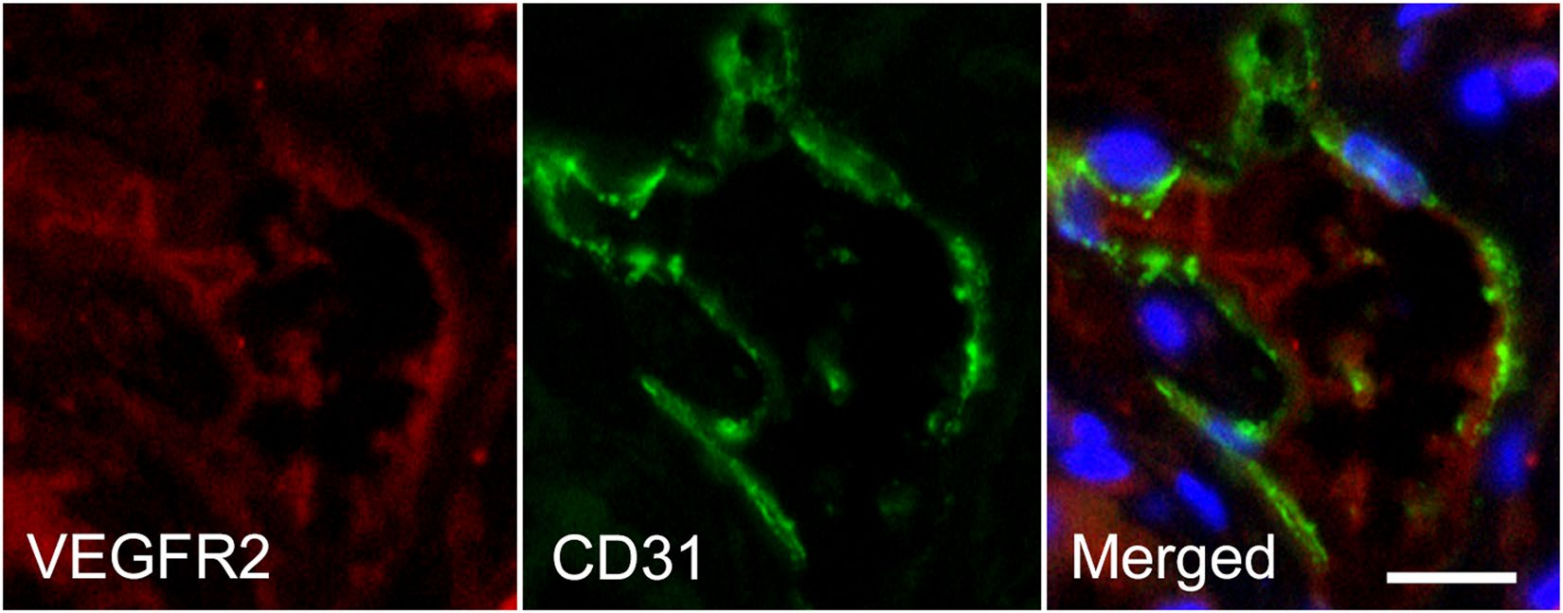
Immunofluorescence staining of human breast cancer (SkBr3) tumor slices for VEGFR2 and CD31. CD31 was expressed on vascular endothelium. Immunofluorescence images of mouse VEGFR2 (red), mouse CD31 (green) and merged VEGFR2- and CD31-stained image (The overlap of red and green fluorescences confirms the colocalization of VEGFR2 with CD31) demonstrated expression of VEGFR2 on endothelial cells of tumor blood vessels in SKBR3 tumors. VEGFR2 was visualized with CY3 dye (red). CD31 was visualized with FITC dye (green). Cell nuclei were stained with DAPI (blue). The scale bar is 20 μm.

**Figure 11:**
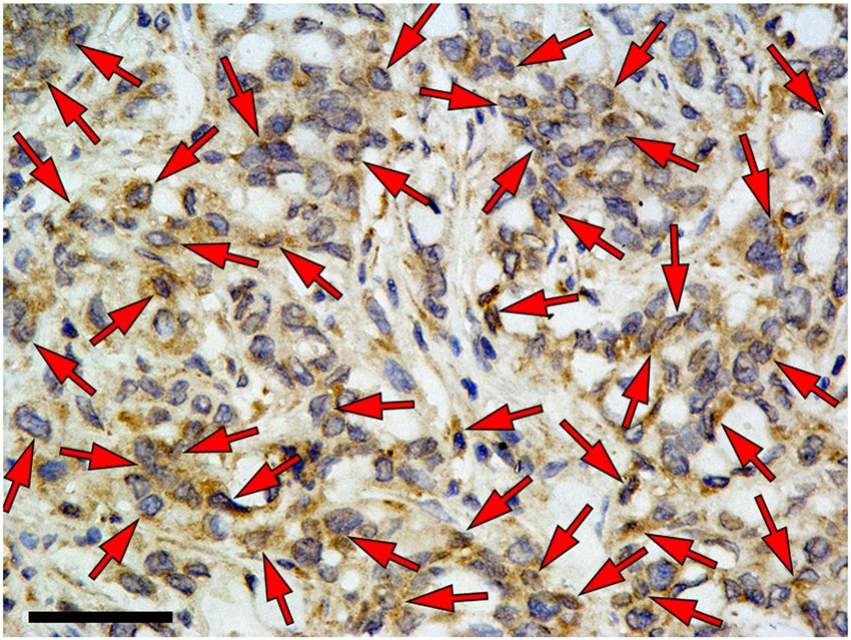
Immunohistochemistry confirmed high HER2 expression on the cell membrane of breast cancer (arrows). Strong dark brown staining was seen on the tumor cell membrane. The scale bar is 50 μm.

After US imaging, tumors were excised and tumor slices were double stained for mouse VEGFR2 and CD31. CD31 was used as a marker of vascular endothelium. Immunofluorescence showed colocalization of VEGFR2 with CD31, confirming the presence of mouse VEGFR2 on endothelial cells within the SKBR3 tumors in our study (Fig. 10). Immunohistochemistry was used to confirm high HER2 expression on the tumor cell membrane of breast cancer. Strong dark brown staining was seen on the tumor cell membrane (Fig. 11).

### *In vivo* toxicity analysis of nude mice

**Figure 12:**
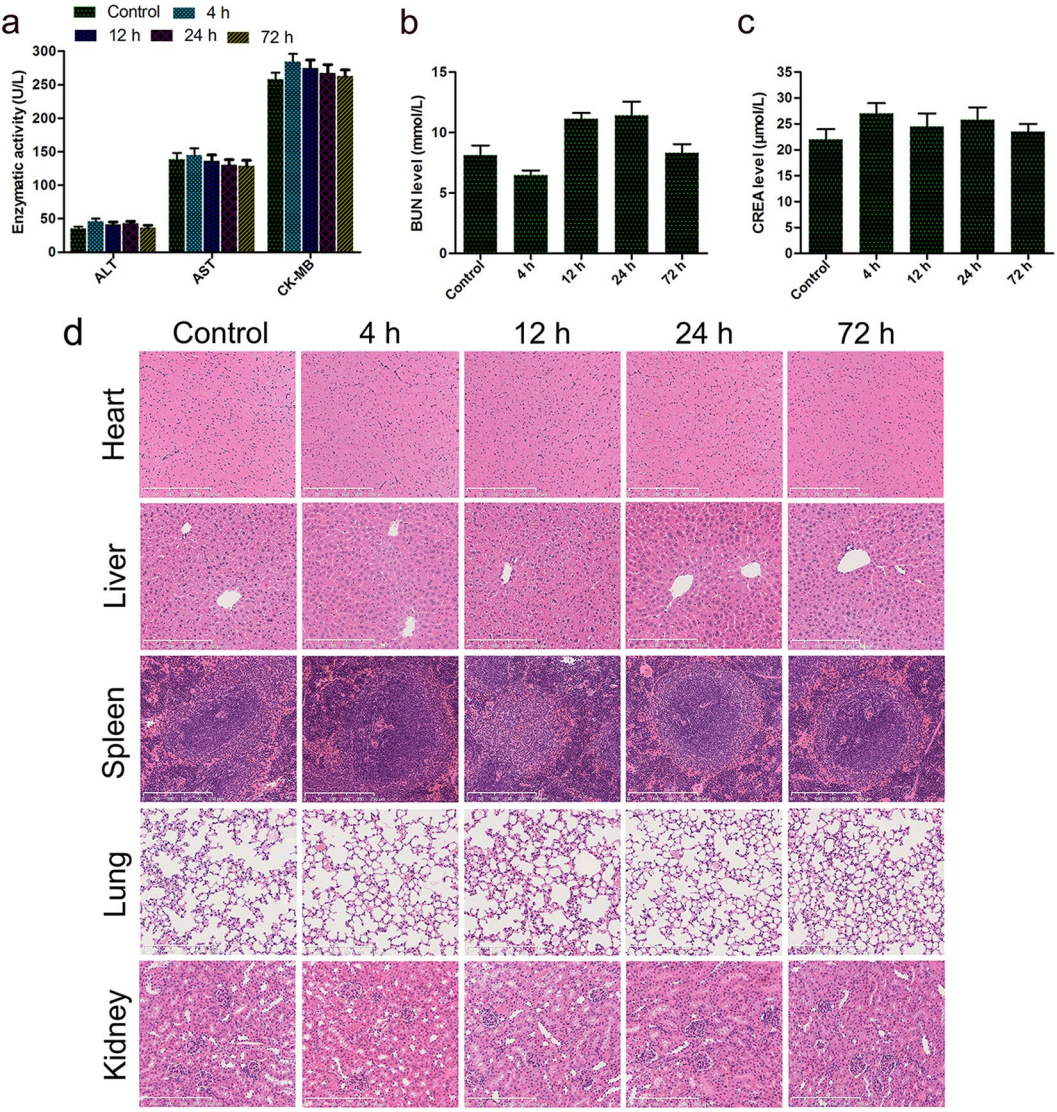
*In vivo* toxicity analysis of breast-bearing nude mice injected with PBS and dual-targeted NBs (4 h, 12 h, 24 h and 72 h). (**a**–**c**) Serum biochemistry data for breast-bearing nude mice injected with PBS and dual-targeted NBs. (**d**) HE staining of organs, including heart, liver, spleen, lung and kidney from mice treated with PBS and dual-targeted NBs. The scale bar is 250 μm.

Finally, the potential *in vivo* toxicity of dual-targeted NBs was further investigated. We observed the behaviors of the mice receiving the injection of dual-targeted NBs or PBS throughout the experimental period and harvested their blood and organs at the endpoint for serum chemistry analysis and HE staining. During the experimental period, no apparent signs of toxic response was observed in the experimental group. A blood biochemistry analysis reflecting the function of major organs was conducted for all the mice at different postinjection time points. As shown in Fig. 12a–c, all biochemistry parameters of mice treated with dual-targeted NBs were within ranges similar to mice treated with PBS. HE staining results shown in Fig. 12d demonstrated that no obvious histopathological abnormalities or tissue damage was noticed in mice receiving treatment in the experimental group. These results suggested that the dual-targeted NBs induced no significant systemic toxicity or other physiological complications *in vivo* at the tested dose. However, further studies would be still needed to systematically evaluate the potential toxicity of dual-targeted NBs at a range of doses.

## Discussion

Previous several studies had developed and tested molecularly-targeted MBs using VEGFR2 for US imaging of the neovasculature of breast cancer^2–4^. It had been proven that VEGFR2-targeted US molecular imaging could improve the diagnostic accuracy of early breast cancer and distinguish breast cancers with different angiogenesis and aggressiveness. Bachawal *et al*.^2^ reported US molecular imaging using VEGFR2-targeted MBs could depict different histological stages of breast cancer development, including mammary hyperplasia, DCIS and invasive breast cancer. Despite the increasing number of published studies on US molecular imaging, a major obstacle for a clinical translation still exists. VEGFR2 single-targeted contrast MBs could not effectively penetrate the leaky tumor vasculature to target the cancer cells, which are only used as blood-pool contrast agents for US molecular imaging and lack of tumor-targeting specificity.

NBs, as novel UCAs, have attracted increasing attention in the field of molecular US imaging for tumors. HER2 is a well-established tumor target that is overexpressed in breast, ovarian and urinary bladder carcinomas, as well as many others^15^. Yang *et al*.^15^ prepared phospholipid-shell and C_3_F_8_ gas-core NBs and conjugated them with biotinylated anti-HER2 Affibody® molecules for HER2-overexpressing tumor imaging. The results demonstrated the high specificity of NB-Affibody conjugates for HER2-overexpressing breast cancer cells in both *in vitro* and *in vivo* experiments, and also verified the high enhancement afforded by NB-Affibody conjugates as a UCA. Although the newly prepared nanosized NB-Affibody conjugates were observed to be a novel targeted UCA for efficient and safe specific molecular imaging, the sensitivity of HER2-targeted UCAs for breast cancer detection was still not high enough since the positive expression rate of HER2 in breast cancer was lower.

In our study, we developed a novel dual-targeted US imaging agent with C_3_F_8_-filled PLGA NBs that attached to VEGFR2 and HER2 and compared the targeting specificity and the resultant US enhancement of dual-targeted NBs with that of single-targeted NBs in VEGFR2-positive and HER2-positive cells and a murine model of breast cancer. The mechanism of formation of air-filled PLGA NBs may be explained by the two following reasons: Firstly, the solvent diffusion-evaporation from the organic phase to the external aqueous phase results in the formation of NBs^30^. Secondly, the added camphor as sublimable porogens is causing voids upon removal by freeze drying, and these voids could be filled with echo-producing gas^31^. In our study, C_3_F_8_ gas, a hydrophobic and dense bioinert gas, was introduced to enhance backscattered signals. Anti-HER2 and VEGFR2 monoclonal antibodies were covalently linked to the PLGA nanobubble surface using a carbodiimide technique. The use of the NHS/EDC as an activator could activate the large number of carboxyl on the PLGA surface, and the conjugation reaction of PLGA NBs with antibodies formed amide bonds between the activated carboxyl groups on the nanobubble surface and the amine groups in the antibodies.

It was also found that dual-targeted PLGA NBs had the ideal particle size range and the stability was better. The real size of an antibody molecule is only about 10 nm^32^. Therefore, the addition of antibody had not changed dramatically the size of PLGA NBs due to the small size of the antibody. In tumors, vessel-wall structure is abnormal with wide interendothelial junctions, an abnormally thick or thin basement membrane, large numbers of fenestrae and transendothelial channels formed by vesicles. Hashizume *et al*.^33^ reported that blood vessels in MCa-IV mouse mammary carcinomas, which were known to be unusually leaky with high leakiness, had a functional pore size of 1200 to 2000 nm. They also confirmed pore sizes of less leaky Shionogi mammary tumors varied from 200 to 380 nm. However, there had been few reports on the pore size on vessels of SKBR3 mammary tumors. The mean diameter of the dual-targeted NBs prepared in our present study was 230.2 ± 58.5 nm. This small size of dual-targeted PLGA NBs could effectively penetrate the leaky tumor vasculature to target the cancer cells, since the fenestrate openings of typical tumors are within the range of 400 nm–600 nm^13^. Therefore, the size distribution of the dual-targeted NBs is appropriate for the retention within SKBR3 tumors due to the enhanced permeability and retention effect.

The qualitative target behaviors of the single- and dual-targeted NBs to VEGFR2 and HER2 were visually identified by LSCM images, and the FCM results provided further evidence that both single-targeted and dual-targeted NBs had high targeting abilities of recognizing and conjugating with the target cells. No obviously statistical difference was found in the specific binding rate to the cells between single-targeted group and dual-targeted group, which might be ascribed to only one target receptor expression in SKBR3 or SVR cells. The capability of targeted PLGA NBs as a contrast agent for US imaging was also assessed *in vitro*. Our results demonstrated that the non-targeted PLGA NB_S_ were able to be used as a contrast agent for efficient US imaging. Moreover, NB_D_ showed no visible difference in the echo signal compared with single- or non-targeted NBs, indicating that the back scattering of the NBs generated from the core-shell structure and the surface-linked antibodies did not weaken the imaging effect. Enhanced echogenicity in the gel was also detected in the SKBR3 cells (high HER2 expression) and SVR cells (high VEGFR2 expression) after incubation with targeted PLGA NBs. Furthermore, the enhanced ultrasonic appearance was only found in SKBR3 and SVR cells, and not in MDA-MB-231 and 4T1 cells that had low expressions of HER2 and VEGFR2. This finding, combined with the confocal imaging and FCM results, suggested that the targeted NBs were the effectors of the observed ultrasonic enhancement. The results of our *in vitro* study showed that the single- or dual-targeted PLGA NBs could specially bind to corresponding target cells and had better effects of target enhancement.

Contrast agents binding to more than one molecular marker may be advantageous over single-targeted contrast agents by increasing the number of NBs attached at sites of tumor angiogenesis and tumor cells. In our *in vivo* study, we demonstrated that dual-targeted NBs that were dually targeted to both VEGFR2 and HER2 led to higher level of US imaging signals than either of the single-targeted NBs at sites of tumor angiogenesis or tumor cells in tumor-bearing mice. The results suggested that the dual-targeted PLGA NBs had the stronger targeting ability, which was receptor-mediated through the combination of dual-targeted NBs and both HER2 on the SKBR3 tumor cells and VEGFR2 on the tumor vascular endothelial cells. A synergistic interaction between the two binding ligands and the enhanced permeability and retention effect of nanoscale contrast agent could produce more specific and effective US molecular imaging for the detection of breast cancer. Dual-targeted UCA may enhance the specific US signals from tumor neovasculature and tumor cells, hence, may not only facilitate cancer detection, but also cancer treatment monitoring *in vivo*.

In our study, two different imaging systems were adopted to investigate the targeted contrast-enhanced capability of dual-targeted NBs in breast tumor mice model. *In vivo* imaging experiment using 22 MHz linear transducer collected and analyzed 120 imaging frames in B-mode and displayed the difference in video intensity from subtraction of the pre- and post-destruction images. Meanwhile, the dedicated small animal US imaging system with a 50 MHz probe could digitally record dynamic character images in contrast mode and overlay the US enhanced signals (green color) on the background. In our imaging experiment using 22 MHz probe, imaging was suspended for 6 min after injection of targeted NBs. This time allowed binding and retention of targeted NBs while awaiting wash-out of the unbound contrast agent. Then, a continuous (16-MHz) high-power US destruction sequence was applied (a MI of 0.3) for 5 s to destroy the NBs within the beam elevation. The gray-scale intensity-over-time curves obtained by the imaging system using 50 MHz probe showed that the signal intensity in the tumor region had a significant rise about 6 min after administration of dual-targeted NBs, and then maintained at a steady level. This result indicated that the 6-minute waiting period during which the targeted NBs were allowed to bind and retain within the tumors was accurate and the targeted NBs had combined with the target in the body. During a period of 8 min after the administration of dual-targeted NBs, only 1000 imaging frames could be collected by our present imaging instrument, which were used to generate the gray-scale intensity-over-time curve and record the dynamic targeting enhancement process, so that the accurate *in vivo* imaging time of the dual-targeted NBs could not be evaluated.

When the bubbles were modified from micron to nanosize, the bubble size would affect the backscattered acoustic signals and nonlinear oscillations. The bubble size is inversely related to backscattered acoustic signals and nonlinear oscillations. Except for the bubble size, composition of the core–shell structure, acoustic pressure amplitudes and acoustic excitation frequencies in the sound field would also affect backscattered acoustic signals or nonlinear oscillations. In a word, backscattered acoustic signals or nonlinear oscillations that the contrast agent bubbles generate depend on the bubble characteristics in combination with the US field applied. In the our present study, a 22 MHz US probe was used to evaluate the imaging effects of the dual-targeted NBs with the mean diameter of 230.2 ± 58.5 nm. Our *in vitro* results demonstrated that the dual-targeted NBs were able to be used as a contrast agent for the efficient US imaging, and the length of imaging time could meet the requirement of clinical contrast-enhanced US. In the most previous studies^4,17,20^, a 40 MHz high frequency probe was usually used for *in vivo* US molecular imaging, which was ideal for small sample and small animal imaging. However, a 22 MHz high frequency probe for human was also used in our *in vivo* study. It could also be better to display the two-dimensional B-mode gray-scale US image of breast tumor and obtain the molecular imaging signals attributable to targeted UCA adherent to vascular endothelial and cellular biomarkers. Our results showed that the nanosized targeted UCA prepared in this study could be more suitable for human ultrasonic probes and imaging system, providing potential possibility for the conversion of *in-vivo* findings into human subject applications.

*In vivo* special affinity of dual-targeted PLGA NBs to both VEGFR2 and HER2 was further confirmed using a fluorescence imaging system of small animals. The results of our study showed the tumor fluorescence signals of mice injected with NB_D_ significantly increased with time post-injection, suggesting that VEGFR2 and HER2 could be used as important molecular targets of UCAs for the identification of breast cancer. Moreover, the dual-targeted nanosized UCA that we had prepared specifically accumulated in the target area, which could be helpful to achieve the targeted US molecular imaging of breast cancer.

In some previous studies, in order to ensure a direct intraindividual comparison between video intensities derived from the targeted and non-targeted UCAs, the different types of MBs were injected consecutively in the same animals during the same imaging sessions^4,17,20^. To further obviate any bias from the order of MBs injections and to minimize interactions between the MBs types, the different MBs were administered in random order and a delay of 30 min between the MBs injections was used, since most of the MBs were thought to be cleared from the mouse vasculature within 30 min after intravenous injection. In our study, *in vivo* lifetime of the dual-targeted NBs could be indirectly evaluated using a fluorescence imaging system of small animals. As time went on, the tumor fluorescence signal of mice injected with dual-targeted NBs gradually increased, peaked at 30 min. Then, fluorescence signal of the dual-targeted NBs gradually reduced and still had not completely disappeared until 60 min. In our study, the dual-targeted NBs were prepared with a biodegradable polymeric shell composed of PLGA. A liquid chromatography-tandem mass spectrometry assay could be used to determine PLGA concentrations in rat plasma. By measuring PLGA concentrations, it was possible to accurately estimate *in vivo* lifetime of the dual-targeted NBs. The dual-targeted NBs remained in the tumor area for a longer period because they exhibited the enhanced permeability and retention effect and their potentials of active targeting at the tumor cells, which was not shown by targeted MBs. Another possible explanation for this phenomenon might be that the dual-targeted NBs were “smarter” to escape the capture by liver or spleen than targeted MBs due to their small size. It was indicated that the interval of 30 min after injection of targeted NBs was not enough long and effective to separate the targeted contrast agent from the receptors. In the current study, we had more experimental groups, and the mortality rate of mice bearing tumors might be significantly increased since the same animal repeatedly received the contrast agents and long anesthesia. Moreover, considering the target accumulation time of the dual-targeted NBs and avoiding a decrease in the specific binding ability of the receptors caused by repeated injections, therefore, the comparative study of the different targeted NBs for the US molecular imaging was not carried out in the same animal, but in the different experimental animal groups. In addition, although previous studies administered the different types of MBs in random order, they still could not exclude some confounding interactions from repetitive contrast agent administration within the same animal, which might have influenced the absolute values of video intensities obtained in their studies^4,17,20^.

Immunofluorescence staining of human breast cancer (SkBr3) tumor slices for VEGFR2 and CD31 confirmed the colocalization of VEGFR2 with CD31. Moreover, the immunohistochemistry staining image indicated high HER2 expression on the cell membrane of breast cancer. These findings provided histopathological evidences for *in vivo* dual-targeted US molecular imaging.

The following limitations of the study need to be addressed. Firstly, the comparative study of the different targeted NBs for the US molecular imaging was not carried out in the same animal, but in the different experimental animal groups. Therefore, in the breast cancer tissues of different nude mice, the imaging signal derived by using targeted contrast-enhanced US depends on the number of tumor blood vessels and tumor cells, the expressions of the receptor molecules in tumor tissues and the positioning of the transducer on the tumor, which limits the repeatability of studies. Secondly, in our study, although there was a certain increase in video intensity after administration of dual-targeted NBs compared with single-targeted and non-targeted NBs, the echo characteristics of dual-targeted UCA based on PLGA NBs in *in vivo* experiments of mice bearing SKBR3 tumors still need to be improved due to weaker ultrasonic reflection ability, smaller cavity structure and shorter imaging time. A previous study by Ke *et al*.^34^ had developed a novel multifunctional UCA based on gold-nanoshelled microcapsules by electrostatic adsorption of gold nanoparticles as seeds onto the polymeric microcapsule surfaces. Their results showed that gold-nanoshelled polymeric microcapsules could be used as a theranostic agent for simultaneous diagnosis and treatment of tumors by utilizing their enhanced US imaging capabilities and photothermal effects. Further studies are needed to evaluate whether the formation of gold nanoshell on the surface of the PLGA NBs might increase the echo signals of targeted UCA, improving the US contrast performance. Thirdly, in our present study, although *in vivo* special affinity of dual-targeted PLGA NBs to both VEGFR2 and HER2 was confirmed using a fluorescence imaging system of small animal, *in vivo* blocking of molecular targets was not performed to further validate our results. Additional studies in which the video intensities of the targeted inhibition group pre-treated with free antibodies are compared with those of the targeted group are warranted and will further help confirm binding specificity of dual-targeted PLGA NBs to both VEGFR2 and HER2. Fourthly, the amounts of antibodies which conjugate on each PLGA NBs need to be measured to further confirm the validity of the connections between the NBs and antibodies. Fifthly, the frozen section of tumors should be prepared, and the observation for the distribution of NBs within tumors is very meaningful to further verify the accuracy of the dual-targeting. Lastly, further study is still required to investigate whether dual-targeted US molecular imaging could differentiate different histological stages of breast cancer and allow highly accurate detection of both early breast cancer and its precursor lesion DCIS.

In conclusions, a novel dual-targeted nanosized UCA directed at both VEGFR2 and HER2 is developed, and the feasibility of using dual-targeted PLGA NBs to enhance ultrasonic images is demonstrated *in vitro* and *in vivo*. This may be a promising approach to target biomarkers of breast cancer for two site-specific US molecular imaging.
